# The Body as a Battlefield: Identity Development and Psychosomatic Expression in Eating Disorders Across Childhood and Adolescence

**DOI:** 10.3390/children12111465

**Published:** 2025-10-29

**Authors:** Giuseppe Marano, Daniele Napolitano, Esmeralda Capristo, Gianandrea Traversi, Osvaldo Mazza, Eleonora Gaetani, Marianna Mazza

**Affiliations:** 1 Unit of Psychiatry, Fondazione Policlinico Universitario A. Gemelli IRCCS, 00168 Rome, Italy; 2 Department of Neurosciences, Università Cattolica del Sacro Cuore, 00168 Rome, Italy; 3 CEMAD—Fondazione Policlinico Gemelli IRCCS, 00168 Rome, Italy; daniele.napolitano@policlinicogemelli.it; 4 Department of Translational Medicine and Surgery, Fondazione Policlinico Universitario A. Gemelli IRCCS, Università Cattolica del Sacro Cuore, 00168 Rome, Italy; 5 Department of Medical and Surgical Sciences, Fondazione Policlinico Universitario A. Gemelli IRCSS, 00168 Rome, Italy; 6 Unit of Medical Genetics, Department of Laboratory Medicine, Ospedale Isola Tiberina-Gemelli Isola, 00186 Rome, Italy; gianandrea.traversi.fw@fbf-isola.it; 7 Spine Surgery Department, Bambino Gesù Children’s Hospital IRCCS, 00168 Rome, Italy; 8 Unit of Internal Medicine, Cristo Re Hospital, 00167 Rome, Italy

**Keywords:** identity disturbance, self-concept clarity, objectification, self-discrepancy, interoception, mentalization, anorexia nervosa, bulimia nervosa, binge-eating disorder, childhood, adolescence

## Abstract

**Highlights:**

**What are the main findings?**

- Identity disturbance links early adversity to eating disorders.
- Low self-concept clarity and self-objectification maintain symptoms.

**What is the implication of the main finding?**

- Identity-focused therapies may enhance recovery in young patients.
- Early identity screening can improve prevention and treatment.

**Abstract:**

Background/Objectives: Eating disorders (EDs) frequently emerge during critical stages of childhood and adolescence, when identity development and emotional regulation are still maturing. Disturbances in self-concept clarity and identity integration may transform the body into a symbolic battlefield for autonomy, belonging, and self-worth. This review synthesizes developmental, psychosocial, neurocognitive, and therapeutic perspectives on the role of identity disturbance in EDs. Methods: A narrative review was conducted (2010–2025) using combinations of terms related to identity, self-concept clarity, self-discrepancy, objectification, interoception, and eating disorders (anorexia nervosa, bulimia nervosa, and binge-eating disorder). Results: Findings indicate that identity vulnerability (expressed as low self-concept clarity, heightened self-discrepancies, and self-objectification) mediates the association between early adversity, sociocultural pressures, and ED symptoms. Neurocognitive studies reveal altered self-referential processing, default mode network connectivity, and interoceptive signaling. Clinically, comorbid borderline personality features further exacerbate identity disturbance and complicate recovery. Evidence-based treatments such as enhanced cognitive-behavioral therapy (CBT-E) effectively target core maintaining mechanisms, while adjunctive interventions (mentalization-based therapy, schema therapy, narrative approaches, and compassion- or acceptance-based methods) show promise in addressing identity-related processes and improving outcomes. Conclusions: Identity disturbance provides a unifying framework for understanding why ED symptoms become entrenched despite adverse consequences. Integrating identity-focused approaches with nutritional and medical care may enhance recovery and reduce chronicity in youth. Future research should adopt longitudinal and mechanistic designs to clarify pathways linking identity change to clinical improvement and test identity-specific augmentations to standard ED treatments.

## 1. Introduction

A growing body of evidence suggests that identity disturbance, variously indexed by identity diffusion, confusion, or low self-concept clarity (SCC), is closely linked to the onset and persistence of eating disorders (EDs). Lower SCC prospectively heightens vulnerability to sociocultural appearance pressures and comparisons, paving the way from early adversity to body dissatisfaction and ED symptoms [1,2]. Clinical studies likewise show that, during treatment, improvements in identity synthesis and reductions in identity confusion co-vary with decreases in drive for thinness and body dissatisfaction, underscoring identity processes as dynamic treatment targets [3]. In many patients, the body becomes a primary medium for negotiating who one is and how one should be; control of eating, weight, and shape is drafted into service to manage conflicts about autonomy, belonging, and self-worth. This translation of self-regulatory struggle onto somatic terrain is coherent with self-discrepancy theory, where gaps between actual, ideal, and ought selves fuel shame and agitation that ED rituals promise to regulate [4], and with objectification theory, which links internalized body surveillance and shame to disordered eating [5,6]. Conceptually, the “body-as-battlefield” metaphor captures how these pressures consolidate a fragile sense of self even as medical and psychosocial risks mount; transdiagnostic models further show how overvaluation of shape/weight can fuse with self-worth, entrapping identity within the illness [7]. Converging neurocognitive findings, altered self-referential/default-mode network connectivity, and interoceptive disturbances provide mechanistic support for this identity–body linkage and show partial normalization with treatment [8,9]. Recent evidence suggests an increasing incidence of early-onset anorexia nervosa in preadolescence (<12 years), with unique challenges including growth impairment, delayed puberty, and greater medical instability. These features highlight the developmental specificity of EDs in children and the importance of early detection and intervention [10].

Collectively, these lines of evidence point toward augmenting standard ED care with interventions that explicitly cultivate self-integration and meaning, alongside nutritional and medical rehabilitation [7,11].

Moreover, recent research highlights that early adverse experiences, such as emotional neglect, abuse, or family conflict, can compromise the development of a coherent self-concept, thereby reducing self-concept clarity and increasing vulnerability to sociocultural appearance pressures. Longitudinal data indicate that childhood maltreatment predicts lower self-concept clarity, which in turn mediates the link between early adversity and body dissatisfaction or disordered eating during adolescence [1,2,3]. These findings suggest that impaired identity development may serve as a psychological bridge between early environmental risk and later eating pathology, emphasizing the importance of early relational and developmental interventions.

## 2. Materials and Methods

This work was conducted as a systematized scoping review in accordance with the Preferred Reporting Items for Systematic Reviews and Meta-Analyses extension for Scoping Reviews (PRISMA-ScR).

### 2.1. Search Strategy

A comprehensive literature search was performed between January 2010 and August 2025 across four electronic databases: PubMed/MEDLINE, PsycINFO, Web of Science Core Collection, and Google Scholar.

The following keywords and Boolean combinations were used:

(identity OR “identity disturbance” OR “self-concept clarity” OR “self-discrepancy” OR objectification OR mentalization OR interoception) AND (“eating disorder” OR “anorexia nervosa” OR “bulimia nervosa” OR “binge-eating disorder”) *.

The search was limited to peer-reviewed articles in English involving human participants (with particular attention to subjects aged ≤25 years) or studies explicitly addressing developmental trajectories of eating disorders. Reference lists of eligible papers and recent special issues were also screened manually to identify additional records. The 2010–2025 timeframe was chosen to capture the modern wave of identity-focused research in eating disorders, coinciding with the introduction of standardized measures of identity development (e.g., Assessment of Identity Development in Adolescence or AIDA) [12] and the emergence of the Identity Disruption Model [1]. This period also aligns with the expansion of neurocognitive and psychotherapeutic studies addressing self-concept clarity, mentalization, and embodiment, ensuring comprehensive coverage of contemporary evidence.

### 2.2. Inclusion and Exclusion Criteria

Studies were eligible if they examined identity-related constructs (e.g., self-concept clarity, identity diffusion, self-discrepancy, self-objectification, interoception, or mentalization) in the context of eating disorders; included quantitative, qualitative, longitudinal, neurocognitive, or treatment-based designs; and reported empirical data or systematic synthesis relevant to identity or self-processes.

Exclusion criteria were studies not directly addressing identity or self-related constructs (e.g., purely nutritional or genetic studies); populations outside the ED spectrum (e.g., obesity without ED diagnosis); non-peer-reviewed sources, conference abstracts, and case reports without sufficient data; and articles not in English or unavailable in full text.

### 2.3. Screening and Selection Process

A total of 801 records were identified (databases = 800; other sources = 1). After removal of 231 duplicates, 570 titles/abstracts were screened.

**Figure 1:**
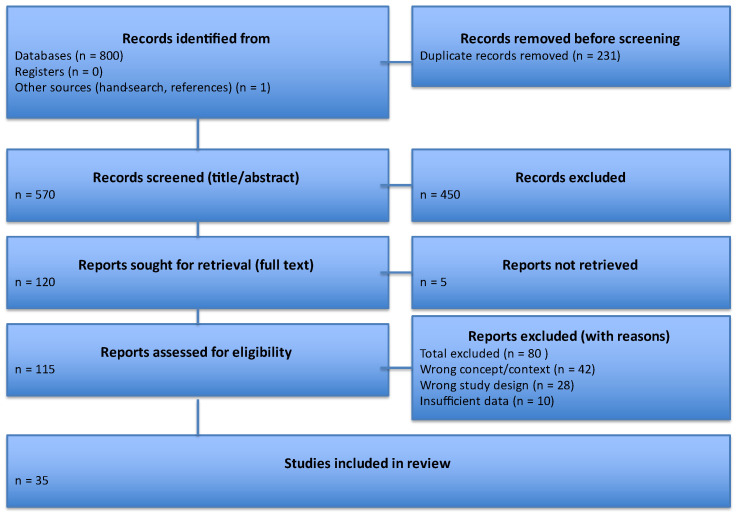
PRISMA 2020 flow diagram of identification, screening, eligibility, and inclusion of studies.

Following exclusion of 450 irrelevant records, 115 full texts were assessed for eligibility; 35 studies met the inclusion criteria and were incorporated into the final synthesis. Reasons for exclusion were as follows: wrong concept or context (n = 42), ineligible study design (n = 28), and insufficient data (n = 10). The entire process is summarized in Figure 1 (PRISMA flow diagram).

### 2.4. Data Extraction and Synthesis

For each included study, the following information was extracted: author(s), year, study design, sample size and characteristics, main constructs assessed, and key findings.

Results were synthesized narratively, integrating developmental, psychosocial, neurocognitive, and therapeutic perspectives. A quantitative meta-analysis was deemed inappropriate because the included studies varied widely in constructs, instruments, diagnostic samples, and outcome metrics. These discrepancies violate the assumptions of statistical homogeneity required for meta-analytic aggregation. Following PRISMA-ScR guidance, a narrative synthesis was therefore selected to allow integration across developmental, psychosocial, and neurobiological dimensions of identity disturbance in eating disorders.

### 2.5. Risk of Bias and Methodological Considerations

Formal risk-of-bias assessment was not performed, consistent with PRISMA-ScR guidance; however, potential sources of bias were acknowledged, including variability in measurement tools, sample composition, and publication bias favoring Western or female-dominant samples.

## 3. Conceptual Frameworks Linking Identity and EDs

Understanding the relationship between identity and EDs requires integrating multiple theoretical perspectives that describe how disturbances in self-structure and self-evaluation manifest. The frameworks reviewed below (developmental identity theory, self-discrepancy theory, and self-objectification theory) share a common focus on the integrity and stability of the self but differ in emphasis: developmental models address the structural process of identity formation and commitment; self-discrepancy theory conceptualizes internal conflict among self-guides as a source of affective distress; and objectification theory focuses on sociocultural embodiment and the externalization of self-worth through appearance. Together, these perspectives converge on identity disturbance as a unifying construct while illuminating distinct cognitive, emotional, and social mechanisms that sustain disordered eating.

### 3.1. Developmental Identity Theory and Self-Concept Clarity

Eriksonian and neo-Eriksonian theories frame identity formation as a normative, staged task in which adolescents move through exploration toward commitments that organize values, goals, and social roles [13]. Marcia’s identity status paradigm operationalizes this process as achievement (high exploration, high commitment), moratorium (high exploration, low commitment), foreclosure (low exploration, high commitment), and diffusion (low exploration, low commitment), providing a widely used structural lens on adaptive versus vulnerable trajectories [14]. Meta-analytic and longitudinal work shows that adolescence is the critical window in which movement from diffusion/foreclosure toward achievement typically occurs; failure to do so co-occurs with greater internalizing symptoms and dysregulation [11,15]. Converging epidemiological reviews also indicate that the peak incidence of eating disorders (EDs) falls in mid-adolescence, with hints of a shift toward earlier presentation in recent cohorts, underscoring the developmental intersection of identity work and ED risk [10,16,17]. Within this framework, SCC has emerged as a tractable, dimensional marker of identity functioning: the extent to which one’s self-beliefs are clearly and confidently defined, internally consistent, and stable over time [18]. SCC is reliably measured with the self-concept clarity scale and differentiates the “knowledge structure” of the self from its evaluative tone (self-esteem). Low SCC indexes a fragmented or incoherent self-structure and has been linked cross-sectionally to emotion dysregulation and psychopathology, features that frequently precede or accompany EDs in youth. A growing empirical literature situates SCC directly within sociocultural pathways to body image and disordered eating. The Identity Disruption Model proposes that early adversity undermines SCC, which in turn heightens internalization of appearance ideals and appearance-comparison tendencies; these processes increase body dissatisfaction and disordered eating [1]. Tests of the model in adolescents replicate these pathways: lower SCC relates to stronger thin-/fit-ideal internalization and more comparisons, which in turn relate to greater body dissatisfaction [2].

These findings are in line with the Tripartite Influence Model, where parental, peer, and media pressures operate through internalization and comparison to shape body dissatisfaction and eating pathology [12,19,20]. SCC appears to function as a development-sensitive “gateway” variable: when self-knowledge is diffuse or unstable, adolescents are more permeable to sociocultural appearance pressures, translating identity uncertainty into body-focused regulation strategies (restriction, over-exercise, binge/purge), thereby consolidating a precarious sense of self via the body. To anchor key constructs and measures used in ED research, Table 1 summarizes commonly employed identity, objectification, and self-discrepancy instruments, typical uses, and example references (see Table 1).

### 3.2. Self-Discrepancy Theory

Higgins’ self-discrepancy theory (SDT) proposes that misalignments among the actual, ideal, and ought selves generate distinct negative affects (e.g., shame/dejection from actual–ideal gaps; agitation/guilt from actual–ought gaps) [4]. In EDs, these self–self-mismatches are often organized around appearance and worth, such that attempts to reduce weight, reshape the body, or rigidly control eating operate as identity-regulating rituals that seem to “close” the gap, at least transiently, thereby negatively reinforcing ED behaviors [4,26,27]. Early empirical work linked specific discrepancies to symptom profiles (actual–ideal with body dissatisfaction; actual–ought with anorexic-type attitudes/behaviors), and these effects were observed even when appearance attributes were excluded from discrepancy scoring, implicating structural self-mismatch rather than content alone [26]. More recent transdiagnostic studies show that actual–ideal discrepancies tend to associate more strongly with anorexia nervosa, whereas actual–ought discrepancies relate to bulimia nervosa and binge-eating disorder and to greater purging, binge eating, and global ED severity, supporting SDT mechanisms as cross-cutting maintenance factors [28]. At the body-image level, actual-ideal body size/shape discrepancy (e.g., silhouette difference scores) and implicit indices both predict higher body dissatisfaction and ED symptoms [29,30]. Clinically, desired-weight discrepancy (the gap between goal and current/healthy weight) captures the same process in actionable terms and prospectively predicts greater ED severity and more entrenched cognitions during treatment, particularly among adolescents, even when weight restoration is achieved [31,32]. Collectively, these findings situate self-discrepancy as a mechanism by which rigid ideals and “oughts” about thinness pull behavior toward symptom-maintaining rituals, and they motivate interventions that soften extreme standards, diversify self-definitions beyond the body, and increase flexibility in responding to body-related thoughts and affects.

### 3.3. Self-Objectification Theory and Self-Objectification

Objectification theory proposes that in cultures where bodies, especially girls’ and women’s bodies, are routinely evaluated and sexualized, individuals internalize an observer’s gaze on their own body (self-objectification), shifting attention toward how the body looks over how it feels or functions [5,33]. This stance fosters habitual body surveillance and vulnerability to body shame, which, in turn, elevates risk for disordered eating as a means to manage appearance-based contingencies of self-worth [5,33].

A large empirical base links self-objectification with eating pathology. A moderate association between self-objectification and disordered eating has been found, consistent with the theory’s risk hypothesis [34]. Tests of the “core” mediational pathway show that self-objectification → body surveillance/shame → disordered eating accounts for symptom variance across diverse samples [23,35]. Recent quantitative syntheses further confirm moderate links between self-objectification and both body shame and body dissatisfaction [23,34,35,36]. These processes emerge early and are amplified in digital ecologies. Developmentally, self-objectification is documented in girls and intensifies with age across adolescence [6]. Media contexts that sexualize or idealize appearance increase self-objectification, with meta-analytic evidence linking sexualizing media use to greater self-objectification [24]. In social media settings, appearance-focused engagement (e.g., Instagram use) predicts body concerns via self-objectification pathways [37], and scoping and systematic reviews implicate social media exposure in body image and eating-related harms in youth [25,38]. Body-related social comparison appears to be a proximal mechanism: moderate associations between online comparison and body-image concerns and eating-disorder symptoms have been reported [25]. In clinical practice, objectification processes are relevant across genders and intersect with other risks (e.g., minority stress, perfectionism), but they consistently forecast body surveillance, shame, and restrictive/compensatory behaviors. Assessment that includes self-objectification, body surveillance, and body shame, alongside appearance-comparison tendencies, can help identify identity-relevant maintenance loops. Interventions that reduce habitual body monitoring and appearance-contingent self-worth, disrupt social comparison (especially in image-centric platforms), and strengthen body functionality appreciation may weaken identity regulation through the body and complement standard ED care [23,39].

### 3.4. Eating Disorders-Specific Maintenance Models: The Transdiagnostic Enhanced Cognitive-Behavioral Therapy (CBT-E) Model

Fairburn’s transdiagnostic cognitive-behavioral account proposes that a common set of maintaining processes underpins the persistence of the main eating disorders. Core among these is the overvaluation of weight and shape, which makes control of eating/weight the principal basis of self-evaluation; this, in turn, drives dietary restraint, body checking/avoidance, and related weight-control behaviors. For many patients, additional processes, like mood intolerance, clinical perfectionism, interpersonal problems, and core low self-esteem, further entrench the cycle [7,40,41]. When self-worth is narrowly tied to weight/shape, the illness can crystallize into a provisional identity: “being the eating disorder” organizes daily life and relationships and complicates disengagement, especially in anorexia nervosa [42,43,44]. Empirically, overvaluation functions as a cross-diagnostic maintaining factor. Reviews and longitudinal studies indicate that higher baseline overvaluation predicts greater eating-disorder psychopathology, more frequent binge eating, and poorer treatment outcomes at end-of-treatment and follow-up; reductions in overvaluation can partially mediate improvement [45,46].

Behavioral expressions closely linked to overvaluation (body checking and body image avoidance) show strong associations with ED pathology, supporting their role as maintenance behaviors targeted in specialized, manual-based, transdiagnostic treatment Enhanced Cognitive-Behavioral Therapy (CBT-E) [47]. It usually requires 18 outpatient sessions, but more sessions may be needed depending on the nature of a patient’s illness. The “broad” form of CBT-E adds modules that directly address non-appearance-maintaining factors when present. Mood intolerance (difficulty tolerating intense affect) is linked to binge/purge and compulsive exercise as mood-modulating strategies; clinical perfectionism sustains rigid rules around eating/weight and all-or-nothing responses to minor deviations; interpersonal problems (e.g., criticism, conflict, isolation) precipitate and perpetuate symptom use; and core low self-esteem keeps self-worth chronically contingent [41,48]. These extensions are often crucial when identity is fused with the illness: broad-form strategies help loosen appearance-contingent self-worth, diversify valued domains, and build alternative sources of identity. In adults with transdiagnostic EDs, CBT-E produced faster early improvement with great efficiency and large gains in self-esteem [49]. Trials and practice-based studies show CBT-E is feasible and effective across diagnoses, including adolescents, where outcomes are broadly comparable to family-based treatment in non-randomized effectiveness work [50,51]. Collectively, the literature supports the model’s core maintenance loop (overvaluation → restraint/checking → symptom escalation) and the utility of focused vs. broad CBT-E selection based on which additional processes are active, particularly relevant when the ED has become a central self-definition [40,42,52].

**Figure 2:**
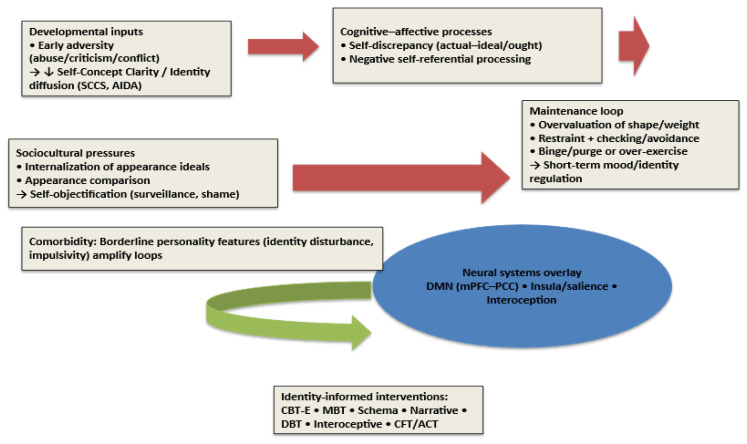
Identity-centered model of eating disorder development, maintenance, and recovery. Abbreviations: ED = Eating Disorder; SCC = Self-Concept Clarity; SCCS = Self-Concept Clarity Scale; AIDA = Assessment of Identity Development in Adolescence; DMN = Default Mode Network; mPFC = Medial Prefrontal Cortex; PCC = Posterior Cingulate Cortex; CBT-E = Enhanced Cognitive-Behavioral Therapy for Eating Disorders; MBT = Mentalization-Based Therapy; DBT = Dialectical Behavior Therapy; CFT = Compassion-Focused Therapy; ACT = Acceptance and Commitment Therapy; rTMS = Repetitive Transcranial Magnetic Stimulation; tDCS = Transcranial Direct Current Stimulation; BPD = Borderline Personality Disorder.

Bringing these frameworks together, Figure 2 presents an identity-centered model linking early adversity, identity disruption, sociocultural pressures, and neurocognitive systems to ED maintenance and recovery pathways. The figure integrates developmental, sociocultural, cognitive-affective, and neurobiological levels to depict how identity disturbance sustains ED pathology. Early adversity undermines self-concept clarity and identity integration, increasing susceptibility to internalization of appearance ideals and appearance-based social comparison, which foster self-objectification. These pressures converge on a cognitive-affective hub characterized by self-discrepancies (actual-ideal/ought) and negative self-referential processing. A neural overlay highlights alterations in default-mode (medial prefrontal–posterior cingulate) and interoceptive/salience (insula) networks that likely scaffold these self-processes. On this substrate, a maintenance loop emerges: overvaluation of shape/weight promotes dietary restraint and body checking/avoidance, which precipitate binge/purge or over-exercise; these behaviors provide short-term mood/identity regulation and feed back to strengthen overvaluation and identity diffusion. Comorbid borderline-personality features (notably identity disturbance and impulsivity) can amplify the loop. The model also indicates leverage points for recovery: CBT-E for overvaluation/behaviors, Mentalization-Based Therapy (MBT) for self-mentalizing, schema and narrative approaches for self-structure and re-authoring, Dialectical Behavior Therapy (DBT)-informed skills for affect/impulse regulation, interoceptive work for body mistrust, and compassion/values-based methods to broaden non-appearance self-worth.

## 4. Developmental and Psychosocial Pathways

### 4.1. From Early Adversity to Identity Disruption, Sociocultural Pressures, Body Dissatisfaction, and ED Symptoms

Evidence for an “Identity Disruption” pathway has accumulated in both young-adult and adolescent samples. In the original Identity Disruption Model, early adverse experiences (e.g., family conflict, abuse) predicted lower SCC; lower SCC, in turn, heightened internalization of appearance ideals and appearance-focused social comparison, which then predicted body dissatisfaction and disordered eating/exercise [1]. Replications in adolescents show the same serial pattern—early adversity → lower SCC → greater internalization/comparison → higher body dissatisfaction—across two independent cohorts, extending the model to the key risk window for ED onset [2]. Experimental/serial-mediation work also supports the specific sequence whereby early adversity undermines SCC, which then fosters thin-ideal internalization and body dissatisfaction [53]. Taken together, these findings position identity functioning (indexed by SCC) as both a mediator of early risk and a leverage point for prevention that may buffer against sociocultural pressures. In adolescence, identity disruption processes are amplified by the digital environment, where social media platforms increase appearance-based comparisons and internalization of unrealistic ideals. Adolescents show greater vulnerability than adults to body dissatisfaction driven by online peer comparison, underscoring the need for developmental prevention programs [25,38].

### 4.2. Sociocultural and Developmental Influences

While most identity-based models of EDs have been developed in Western, individualistic contexts, increasing evidence highlights that sociocultural pressures to conform to body ideals manifest differently across cultures. In individualistic societies, thinness and appearance are often tied to self-determination, autonomy, and personal achievement, whereas in collectivistic cultures, body ideals are more likely to reflect social harmony, respectability, and moral self-regulation. Comparative studies have shown that exposure to globalized media may “hybridize” these ideals, leading to internal conflicts between traditional relational values and modern appearance-based self-worth [54,55]. Moreover, digital platforms amplify transnational beauty norms that increasingly shape identity formation among adolescents regardless of cultural origin. These findings suggest that identity disturbance may arise not only from internal discrepancies but also from the clash between local cultural identity scripts and globalized body ideals. Addressing cultural context is therefore essential to understanding the diverse trajectories of eating-disorder vulnerability worldwide.

Recent studies emphasize that digital media exposure and social comparison processes amplify body image concerns across diverse cultural settings, reinforcing the need for culturally sensitive models of identity development [55].

### 4.3. Identity Development Status and Clinical Course

Identity impairment is common in adolescent anorexia nervosa (AN) and appears clinically consequential. In inpatient adolescents with AN, greater disturbance in identity development (higher AIDA “diffusion”) at admission predicted a slower trajectory of weight gain over the first 10 weeks, independent of initial Body Mass Index (BMI), suggesting identity dysfunction as a severity marker and treatment target [21]. Across ED diagnoses, personality/identity functioning shows systematic differences: in a multicenter study, patients with AN-restricting subtype displayed relatively better overall personality functioning than AN-purging and bulimia nervosa groups, highlighting clinically relevant stratification along identity/self-regulatory capacities [56]. Longitudinal treatment data indicate that identity functioning improves alongside ED symptom reductions; identity confusion decreases while identity synthesis and adaptive identity processes increase during treatment, with concurrent links to decreases in drive for thinness and body dissatisfaction [57]. Complementary evidence suggests identity functioning differs by illness/recovery stage in AN, consistent with a partly state-dependent contribution that interacts bidirectionally with symptom change [44].

### 4.4. Shame, Self-Blame, and Hostile Self-Attitudes

Identity-related self-evaluative emotions are tightly coupled to ED severity and course. Using the Structural Analysis of Social Behavior (SASB) self-image model, low self-love/acceptance and high self-blame correlate with greater ED symptoms across diagnostic groups; in some subgroups (older Bulimia Nervosa-BN), self-hate also contributes [58]. Prospective data underscore prognostic weight: in a 9-year follow-up, baseline self-blame was the only significant predictor of diagnostic remission, and higher initial self-blame forecasted lower odds of remission [59]. Shorter-term outcomes likewise favor a more accepting self-relation: higher self-love and lower self-attack/self-blame at baseline predicted better 12-month outcomes [58]. Converging meta-analytic and longitudinal evidence points to self-criticism (and self-critical perfectionism) as a transdiagnostic driver of disordered eating, with protective roles for self-compassion [60]. These findings dovetail with self-discrepancy/objectification accounts and reinforce targeting self-blame/hostile self-attitudes within identity-focused ED care.

## 5. Neurocognitive and Neurobiological Correlates of the “Self” in EDs

### 5.1. Self-Referential Processing and the Default Mode Network (DMN)

Across cognitive tasks that ask individuals to evaluate trait words about themselves, patients with AN show more negative self-evaluation, endorsing and remembering negative self-descriptors more readily and quickly than positive ones, consistent with clinical reports of low self-esteem and rigid self-standards [61]. At the neural level, this self-referential negativity is in line with atypical intrinsic connectivity in large-scale networks central to self-processing, particularly the DMN (medial prefrontal cortex and posterior cingulate/precuneus). Resting-state studies show that acute AN is often characterized by widespread underconnectivity and altered network topology; importantly, some longitudinal work suggests that these abnormalities only partially normalize, or even persist, after weight restoration [9,62]. Systematic reviews of resting-state fMRI in EDs converge on DMN alterations together with changes in executive-control and salience circuits implicated in self-evaluation and rumination, though findings vary by age, illness stage (underweight vs. weight-restored), and analytic method [63,64]. Older and recent studies also point to mixed patterns (hyper- or hypo-connectivity) in recovered samples, suggesting state-trait interactions and heterogeneity [65]. In other words, self-referential cognitive bias and DMN dysconnectivity provide converging evidence for a neurocognitive “self” signature in EDs that may improve with treatment but not uniformly normalize [9,61,62,63,64].

### 5.2. Interoception and Insular Cortex

Interoception, described as the sensing and interpretation of internal bodily states, has become a key translational construct across EDs. Recent research indicates altered interoceptive processing across diagnoses, with aberrant activation and connectivity of the insular cortex and broader salience/interoceptive network; these disturbances may contribute to disturbed body ownership, altered awareness of hunger/satiety, and conflict between internal signals and self-standards [66,67]. Contemporary network models position the anterior insula as a hub that integrates interoceptive signals, salience detection, and network switching between DMN and executive systems, mechanisms that map well onto ED phenomenology (e.g., heightened body-focused salience, difficulty shifting from self-rumination to goal-directed control) [68]. Insula-centered interoceptive dysfunction can therefore be interpreted as a plausible bridge between identity-related self-evaluation and embodied symptoms (appetite suppression, fullness intolerance) [66,67,68]. Recent neuroimaging studies confirm alterations in the DMN and interoceptive circuits underlying self-referential processing in eating disorders [69,70]. These findings extend prior evidence of altered insular and medial prefrontal activation, suggesting that impaired neural integration of self-related signals may contribute to identity fragmentation.

### 5.3. Body-Image Processing Networks

Quantitative syntheses and recent reviews identify distributed contributions from paralimbic (insula, cingulate), parietal (inferior parietal lobule/precuneus), temporal, and occipital regions to body-image disturbance in AN/BN, consistent with multisystem models of self and embodiment. Activation likelihood estimation and systematic reviews highlight that perceptual, affective, and cognitive components of body image map onto partially distinct networks: parietal/precuneus for perceptual body representation, insula/amygdala and prefrontal regions for affective/evaluative dimensions, and broader fronto-temporo-occipital circuits for cognitive self-appraisal [71,72,73,74]. These findings support the view that “body as battlefield” dynamics are neurally instantiated across interoceptive-affective-cognitive systems rather than localized to a single “body-image center” [71,72,73,74,75].

## 6. Clinical Phenomenology Across Diagnoses

### 6.1. Anorexia Nervosa (AN)

A recent narrative review argues that identity disturbance may be central to AN, shaping both illness maintenance and recovery [76]. Phenomenologically, many patients describe “becoming the disorder”, self-worth fusing with thinness/control such that relinquishing symptoms feels like a loss of identity [42,52]. Qualitative work on externalization similarly shows patients negotiating whether AN is part of the self or an alien intruder, with treatment gains often tied to re-differentiating the self from the illness [52,77]. Clinically, these findings support addressing identity reconstruction and valued-role diversification alongside weight restoration to reduce the experience of identity voids at symptom reduction [42,76].

### 6.2. Bulimia Nervosa (BN) and Binge-Eating Disorder (BED)

In BN and binge-eating disorder (BED), SCC deficits correlate with global ED severity and impulsivity, suggesting that binge/purge or binge/restrict cycles can operate as short-term identity regulators (numbing, self-punishment, self-soothing) that consolidate a negative self-schema [78,79]. Mechanistically, objectification processes amplify these loops: self-objectification → body surveillance/shame → disordered eating shows robust, meta-analytic support, and recent syntheses confirm moderate associations with body shame/dissatisfaction [23,36]. Contemporary work further ties reward sensitivity and inhibitory-control alterations to binge eating, aligning with impulsivity findings and offering targets for intervention [80]. Together, SCC deficits, impulsivity/reward dyscontrol, and objectification-driven shame/comparisons delineate a self-regulatory loop in BN/BED that can be addressed by integrating identity-focused, emotion-regulation, and comparison-disrupting strategies with standard care.

### 6.3. Comorbidity with Borderline Personality Features

EDs frequently co-occur with borderline personality disorder (BPD) traits, including identity disturbance, and this comorbidity predicts greater self-harm risk and poorer outcomes [52,81]. Population-based/large-sample analyses show patterned links between specific BPD symptom dimensions and ED subtypes (e.g., affective instability/impulsivity with BN/BED), even after accounting for other psychopathology [82]. Within ED samples, disturbed identity uniquely predicts Cluster-B pathology, underscoring identity as a cross-cutting liability [83]. In line with this, emerging guidance recommends integrated care frameworks (e.g., augmenting CBT-E or FBT with DBT-informed modules, crisis planning, and staged goals) when EDs co-occur with clinically significant BPD features [84].

### 6.4. Childhood-Specific Clinical Aspects

In children, EDs often manifest with distinctive age-specific clinical features that differ from those typically observed in adults. For instance, atypical anorexia nervosa can emerge before puberty, where patients may present with severe medical instability despite maintaining weight percentiles within or above the normal range. This presentation poses diagnostic challenges, as the absence of marked underweight may delay recognition and intervention [85]. Importantly, early-onset anorexia nervosa is frequently associated with growth impairment, pubertal delay, and reduced bone mineral density, which can have long-term consequences on physical and reproductive health if not addressed promptly [10].

BED has also been increasingly identified in preadolescent populations, often co-occurring with obesity and psychosocial impairment, such as low self-esteem, social withdrawal, and heightened risk of bullying [79]. In this age group, binge-eating behaviors may function as maladaptive strategies to regulate affect or cope with interpersonal stress, consolidating negative self-schemas at an early developmental stage [78].

Beyond the core symptoms, pediatric EDs can significantly disrupt neurodevelopmental trajectories. Malnutrition and dysregulated eating behaviors during critical windows of growth may impair cognitive maturation, executive functioning, and emotional regulation capacities [51]. Furthermore, disturbances in bone health and neuroendocrine function are particularly concerning in childhood, as they may compromise peak bone mass acquisition and increase lifetime vulnerability to osteoporosis [16].

Table 2 summarizes the distinctive clinical features of eating disorders across childhood, adolescence, and adulthood. This comparative overview underscores the unique medical, psychosocial, and therapeutic considerations relevant to pediatric populations.

## 7. Treatment Implications: Targeting Identity to Augment Standard Care

### 7.1. Evidence-Based ED Treatments and Identity

CBT-E remains the leading transdiagnostic psychotherapy, directly addressing overvaluation of shape/weight, dietary restraint, mood intolerance, clinical perfectionism, and interpersonal maintaining factors [50,86]. Randomized and effectiveness trials show CBT-E’s utility across diagnoses (including adult outpatients and adolescents), yet non-response and chronicity persist for a substantial subgroup [49,87]. Clinically, these harder-to-treat presentations often show identity fusion with the illness, where “being the eating disorder” organizes self-worth and relationships, making symptom relinquishment feel like self-loss, suggesting that explicit work on self-structure and meaning should be integrated alongside standard CBT-E modules [44]. In clinical practice, CBT-E is flexibly tailored according to diagnostic subtype. The “focused” form targets core maintaining mechanisms such as dietary restraint and shape/weight over-evaluation (most relevant for BN and BED), whereas the “broad” form additionally addresses mood intolerance, clinical perfectionism, low self-esteem, and interpersonal difficulties, which are particularly salient in AN and mixed presentations. Treatment goals, therefore, differ in the following: weight restoration and normalization of eating patterns in AN, reduction in binge–purge episodes in BN, and regulation of affective and interpersonal triggers in BED. Therapeutic techniques are correspondingly adjusted (e.g., emotion-tolerance training for mood intolerance, behavioral experiments for self-evaluation, and schema-focused modules for chronic low self-esteem) [40,88].

These subtype-specific applications underscore that CBT-E operates on both cognitive-behavioral and identity-related levels, as changes in self-evaluation and self-definition are central mediators of symptom improvement.

In adolescents, family-based treatment (FBT) remains the first-line approach, emphasizing parental involvement in nutritional rehabilitation and progressive return of autonomy. Evidence shows FBT is superior to individual therapies for weight restoration and remission in younger patients. Integrating identity-focused modules into FBT, such as narrative or values-based work, may further enhance outcomes by supporting the developmental tasks of adolescence [51,89].

### 7.2. Mentalization-Based Approaches

Difficulties in mentalizing, especially self-mentalizing (representing one’s own states), are documented across EDs and relate to alliance and outcome [90,91,92,93]. Early outcome work is mixed but promising: in the NOURISHED multicenter RCT comparing MBT-ED to Specialist Supportive Clinical Management in ED patients with borderline features, high attrition limited inference, yet MBT-ED showed greater reductions in weight/shape concern among completers [94]. Research further supports the feasibility and theoretically coherent change processes [93,95]. In EDs, symptoms are tightly linked to how people understand and regulate inner states, and that is exactly what mentalizing targets. When it is hard to notice, name, and make sense of feelings (“What am I feeling? Why now?”), distress is experienced in the body (“I feel fat”, “my stomach is wrong”) and managed with concrete, controllable actions (restrict, binge, purge, over-exercise). Mentalizing helps translate bodily distress back into mental states, so food/weight no longer has to “carry” emotions. Mentalizing collapses when shame, threat, or conflict spikes. ED behaviors are fast, reliable down-regulators, so they get reinforced. MBT trains patients to spot these “online” mentalizing failures and to restore a curious, reflective stance before problem-solving, weakening the need for symptom use. In addition, many patients mistrust or misread hunger/satiety and confuse fullness with moral failure. Strengthening reflective attention to internal signals (and the thoughts/beliefs wrapped around them) supports re-learning eating and reduces black-and-white rules. EDs often involve hyper- or hypo-mentalizing about others (“They think I am disgusting”, “If I eat, they will reject me”). Improving accuracy and flexibility in reading minds reduces shame, secrecy, and safety behaviors like avoidance and body checking. When the sense of self is fragile, weight/shape and dietary rules provide identity and control. Mentalizing fosters a more coherent self that can hold mixed feelings and goals, making it safer to loosen the disorder’s grip. Overall, MBT for EDs has a clear rationale, preliminary signals of benefit, and a need for larger controlled trials with engagement-enhancing adaptations. Recent feasibility and process studies support the utility of MBT for EDs, particularly in enhancing reflective functioning and emotional coherence [96,97,98].

### 7.3. Schema Therapy

EDs are characterized by maladaptive schemas and modes (e.g., demanding/punitive parent, vulnerable child) that map onto harsh self-standards, shame, and “ED-part” dominance [22,99,100]. Early evidence suggests feasibility and symptom improvement [101,102]. Current multicenter RCTs are underway to compare GST against CBT-E and evaluate cost-effectiveness and mechanisms [103,104]. Clinically, mode work helps patients externalize the ED mode, reduce self-criticism, and strengthen healthy adult self-functions; this is especially relevant for CBT-E non-responders with entrenched self-schema pathology [105]. Beyond symptom reduction, schema therapy targets deeper personality structures that sustain identity disturbance, aiming to foster integration between emotional needs, self-worth, and bodily experience. Techniques such as chair work, imagery rescripting, and limited reparenting can address unmet developmental needs and transform punitive self-relations that perpetuate disordered eating. Preliminary findings indicate that increases in adaptive modes (healthy adult, contented child) parallel decreases in ED severity and self-punitive tendencies, suggesting that identity reconstruction may mediate recovery [99,100,101,102,103].

### 7.4. Narrative Therapy and Identity Reconstruction

Narrative therapy explicitly separates the person from the problem, externalizes the ED voice, and re-authors preferred identity stories. A systematic narrative synthesis and a companion systematic review conclude that evidence is promising yet limited, with most support from qualitative/process data and a need for rigorous outcome trials [43,106]. Clinically, externalization often reduces shame and increases agency, synergizing with identity-focused work in CBT-E/Schema/MBT.

### 7.5. Skills for Emotion/Impulse Regulation: DBT-Informed (DBT-BED)

Because binge/purge cycles frequently regulate affect and self-state, DBT-informed interventions are valuable adjuncts. DBT-BED has resulted in superior outcomes to an active comparison therapy on binge-eating reduction with maintained gains [107]. A 2024 randomized clinical trial of a DBT skills smartphone app for recurrent binge eating showed significant reductions in objective binge episodes and global ED psychopathology at 6–12 weeks, albeit with high attrition, supporting scalable, stepped-care options [108]. Adaptive trial work also suggests DBT can help non-responders to initial CBT [109].

Recent research has demonstrated that mechanism-informed adjuncts can strengthen identity change. Interoceptive exposure targets body mistrust in EDs by systematically evoking feared internal sensations (e.g., hunger, fullness, cardiorespiratory arousal) with response prevention and reflective labeling, so patients learn that sensations are tolerable rather than dangerous. This corrective learning reduces catastrophic beliefs, improves interoceptive accuracy, and diminishes reliance on restrictive, binge-purge, or other compensatory behaviors. Pilot and implementation studies support interoceptive exposure to reduce body mistrust and intolerance of internal cues; family-based interoceptive protocols for adolescents are feasible [110,111,112]. Compassion-focused practices train the soothing/affiliative system to counter overactive threat and self-criticism, which are central in eating disorders. Through techniques like soothing-rhythm breathing, compassionate imagery/letter-writing, and cultivating a “compassionate self”, they reduce shame and body-focused self-attack, improving emotion regulation and engagement with nutrition/exposure tasks. A 2024 RCT found Compassion-Focused Therapy (CFT) comparable or advantageous to CBT in intensive settings [113]; earlier trials and meta-analyses link self-compassion/CFT to reduced binge eating and body-image distress [114,115]. Acceptance and Commitment Therapy (ACT) trials targeting body-image/ED symptoms show benefits and fit identity-reconstruction aims by building non-appearance domains of meaning [116,117]. Reviews and early trials suggest rTMS/tDCS can modulate fronto-control/salience networks linked to self-regulation and craving; effects are modest/heterogeneous and best framed as adjunctive, with identity change still requiring psychotherapy [118,119].

### 7.6. Integration of Identity-Focused Approaches

Beyond disorder-specific models, identity-focused approaches such as schema therapy, narrative therapy, MBT, and compassion-focused interventions can be pragmatically embedded within multidisciplinary eating-disorder care. Clinically, this integration involves initial screening for self-concept clarity or identity diffusion using standardized tools (e.g., Self-Concept Clarity Scale, Assessment of Identity Development in Adolescence); collaborative formulation linking identity themes to symptomatic control of eating or body image; targeted modules addressing identity fragmentation (e.g., life-story reconstruction, chair-work, or relational re-mapping); and monitoring of process and outcome indicators, including SCC change, therapeutic alliance, and symptom reduction. Table 3 summarizes each approach’s principal mechanism, session structure, and measurable outcome variable to guide implementation in real-world settings.

This structured framework bridges research and clinical translation by demonstrating how identity work can be operationalized, evaluated, and integrated alongside established interventions such as CBT-E or family-based therapy.

## 8. Psychodynamic Perspective and Psychodynamic Therapy in EDs

From a psychodynamic perspective, ED symptoms are understood as symbolic, defensive solutions to disturbances in self and affect regulation within attachment relationships: eating, weight, and shape become “concretized metaphors” that manage shame, autonomy-dependence conflicts, and fragile identity when reflective/symbolizing capacities are compromised [120,121,122,123,124]. In fact, patients with AN and BN overutilize more neurotic and primitive defense mechanisms compared with nonclinical subjects [125]. Consistent with this relational view, meta-analytic evidence shows markedly higher rates of insecure attachment across EDs, with implications for alliance and outcome [126]. Contemporary psychodynamic formulations integrate object-relations and mentalizing accounts, proposing that deficits in reflective functioning drive the bodily expression of emotion and that treatment should work in the transference to restore symbolization and epistemic trust [121,122,124,127,128].

Psychodynamic approaches are a natural fit for identity-focused formulations because they work directly with self/other representations, conflicted ideals/thoughts, and the “ED part” of the self as it shows up in transference and relationships. In adults with AN, focal psychodynamic therapy (FPT) has manualized protocols and RCT support: in the ANTOP trial, FPT and CBT-E both outperformed optimized treatment-as-usual; CBT-E produced faster weight gain and symptom relief, whereas FPT showed some longer-term advantages in recovery at 12 months [129,130,131]. In adolescents with AN, adolescent-focused psychotherapy (AFT), a time-limited psychodynamic therapy, was inferior to family-based treatment at the end-of-treatment but outcomes converged for a subset over follow-up, supporting AFT as an alternative when FBT is not feasible/acceptable [89]. For BN, CBT seems superior to psychoanalytic psychotherapy for reducing binge/purge, suggesting that if a psychodynamic route is chosen, it benefits from being structured, time-limited, and symptom-linked [132]. Individual, manualized psychodynamic treatments (e.g., FPT) can be considered viable options, especially for adults with longstanding illness/identity fusion, while group psychodynamic formats need more rigorous, manualized trials [133,134,135]. In an identity-centered care plan, psychodynamic work can differentiate the person from the illness within the therapeutic relationship, elaborate conflicted self-guides and body-based self-regulation, and integrate with CBT-E/DBT modules for active symptom management, particularly useful when comorbid personality traits (e.g., BPD features) complicate engagement and maintenance.

## 9. Discussion: What Is the Future for Research and Care?

Several priorities emerge for advancing identity-informed research and care in EDs. First of all, longitudinal designs should test identity-focused mediation: do within-person gains in SCC prospectively predict reductions in ED symptoms after adjusting for mood/anxiety and exposure to sociocultural pressures? The Identity Disruption Model (early adversity → lower SCC → internalization/comparisons → body dissatisfaction → ED symptoms) provides clear hypotheses now replicated in adults and adolescents [1,2]. Parallel work in treatment settings could link identity change to symptom trajectories [57].

Dismantling/augmentation trials should evaluate the incremental benefit of adding identity-targeted modules (e.g., narrative re-authoring, schema/mode work, values-based identity building) to core CBT-E or family-based approaches. Although CBT-E specifies focused vs. broad pathways, such as perfectionism, mood intolerance, and interpersonal problems [7,40], formal tests of identity-specific add-ons are rare; implementation work underscores the need for modular efficiency and personalization [48,86].

Undoubtedly, measurement requires consolidation. Routine assessment should combine SCC [18] with validated identity-diffusion tools in youth, which discriminate clinical from non-clinical samples and now have cross-cultural adaptations [12,136]. Establishing clinical cutoffs and minimal clinically important differences for these measures in ED populations would enable mechanism-focused trials and benchmarking.

Mechanistic trials should connect neurocognitive change (self-referential/DMN, interoception/insula) with identity outcomes and symptoms. Resting-state and task fMRI reviews/meta-analyses implicate DMN and salience/interoceptive circuitry in AN, with partial normalization after treatment [63]; interoceptive meta-analytic work similarly highlights insula-centered alterations across EDs [66]. Pre-registered studies can test whether improvements in SCC/identity mediate neurobehavioral change (e.g., reduced self-referential negativity) and clinical response [137].

Another important issue is the need for tailored interventions for ED–BPD presentations. Population-based and clinical studies show patterned links between borderline features, especially disturbed identity, and ED diagnoses, with poorer outcomes in comorbid groups [84]. Emerging clinical guidance advocates integrated care (e.g., CBT-E or FBT augmented with DBT-informed modules, crisis planning, and staged identity goals), warranting pragmatic and randomized evaluation [84]. Beyond individual and developmental factors, identity and bodily experience are deeply embedded in cultural contexts. Ideals of autonomy, control, and beauty are socially constructed and vary across cultures, shaping both the meanings attributed to the body and the ways identity is expressed through it. In Western societies, the emphasis on independence and self-determination may foster perfectionistic and objectified body ideals, whereas in collectivistic cultures, the body may serve as a medium for social belonging and moral identity. Recognizing these cultural variations enriches the interpretation of identity-related mechanisms in eating disorders and underscores the importance of culturally sensitive prevention and treatment approaches.

Despite the integrative perspective adopted, several methodological limitations should be acknowledged. The studies included in this scoping review displayed substantial heterogeneity in design, assessment tools, and sample characteristics. Moreover, many relied on self-report measures, which may introduce reporting bias, and only a limited number of longitudinal investigations were available to elucidate causal relationships. These factors should be taken into account when interpreting the findings and planning future research aimed at clarifying the temporal and mechanistic links between identity disturbance and ED trajectories. A further limitation of the current literature is its predominant Western focus. Emerging cross-cultural research reveals that the meaning of body image, autonomy, and self-definition varies markedly across societies. For example, collectivistic frameworks may buffer against appearance-based self-worth by emphasizing interdependence, yet they can also foster conformity pressures and shame-based control of eating behaviors. Integrating cross-cultural perspectives may therefore expand the generalizability of identity-focused models and inform culturally sensitive prevention strategies. Future studies should systematically examine how cultural identity, acculturation, and globalization moderate the link between self-concept clarity and disordered eating.

## 10. Conclusions

The reviewed evidence highlights that addressing disturbances of self-concept and identity is not only theoretically informative but also essential for improving long-term outcomes in ED treatment. Identity disturbance offers a unifying lens on why the body becomes the battleground for autonomy, belonging, and worth in EDs, and why symptoms can feel indispensable despite harm. Developmental and sociocultural evidence links early adversity and sociocultural pressures to ED risk via SCC and identity disruption [1,2]. Theoretical frameworks (self-discrepancy, objectification) explain how rigid ideals/thoughts and self-surveillance embed appearance-contingent self-worth and shame; these processes are borne out meta-analytically [4,5,23]. Converging neurocognitive work implicates DMN/self-referential and interoceptive/insular systems, aligning embodiment with identity-related cognition [63,66]. Standard treatments (e.g., CBT-E) effectively target core maintaining mechanisms [7], but identity-focused augmentation, self-integration, narrative reconstruction, and values-based identity building may enhance recovery and reduce chronicity, particularly where illness identity is entrenched [3]. These considerations highlight the necessity of early detection and intervention during childhood and adolescence, when identity structures are most malleable and treatment may alter long-term illness trajectories [16]. Clinically, this perspective implies the systematic assessment of self-concept clarity, identity diffusion, and self-discrepancy at intake and during treatment as process indicators of change. Incorporating identity-focused modules, such as narrative reconstruction, schema work, or mentalization enhancement, within standard CBT-E or family-based therapy may improve emotional regulation, self-coherence, and long-term recovery. Multidisciplinary services could integrate brief screening tools (e.g., AIDA, SCCS) to identify identity vulnerability early, especially among adolescents exposed to high sociocultural pressures or early relational trauma [1,12,40,88].

From a research standpoint, future studies should prioritize mechanistic designs that test identity change as a mediator of symptom improvement, longitudinal analyses mapping developmental trajectories of self-concept clarity, and randomized controlled trials evaluating identity-focused augmentations. Cross-cultural investigations are also needed to determine whether identity-related constructs and their therapeutic relevance generalize beyond Western populations.

In sum, addressing the self as both a developmental and relational construct provides a coherent framework for understanding, preventing, and treating eating disorders, bridging neuroscience, psychosocial research, and clinical practice.

## Figures and Tables

**Table 1:** Identity-related constructs and measures commonly used in eating-disorder research.

Construct	Instrument (Acronym)	What It Measures/Key Subscales	Format (Items; Response Range)	Typical Populations	Clinical/Research Use in EDs (Example)	Example References
Self-concept clarity	Self-Concept Clarity Scale (SCCS)	Clarity/consistency/stability of self-beliefs	12 items; Likert (typically 1–5)	Adolescents; Adults	Mediator/moderator in the Identity Disruption pathway; track change during treatment	Campbell, Trapnell, Heine, Katz, Lavallee, and Lehman [18]; Vartanian, Hayward, Smyth, Paxton, and Touyz [1]; Vartanian, Nicholls, and Fardouly [2]
Identity functioning (youth)	Assessment of Identity Development in Adolescence (AIDA)	Identity synthesis vs. diffusion (e.g., continuity, coherence)	58 items; Likert	Adolescents (clinical and community)	Severity stratification/prognosis in AN; track inpatient change	Goth, Foelsch, Schlüter-Müller, Birkhölzer, Jung, Pick, and Schmeck [12]; Budde, Haenschel, Herpertz-Dahlmann, and Konrad [21]
Identity processes (modes)	Schema Mode Inventory—ED version (SMI-ED)	Maladaptive/functional modes (e.g., Punitive parent, demanding parent, vulnerable child, healthy adult)	≈100 items; Likert	Adults with EDs	Targets for schema therapy: externalize the ‘ED part’	Simpson, McDonald, and Stewart [22]
Self-objectification	Objectified Body Consciousness Scale (OBCS)	Body surveillance; body shame (and control beliefs)	24 items; Likert	Adolescents; Adults	Mediators to disordered eating: change with comparison/media-literacy interventions	Fredrickson and Roberts [5]; Daniels, Zurbriggen, and Ward (2020) [6]; Schaefer and Thompson [23]
Appearance comparison	Physical Appearance Comparison Scale (PACS/PACS-3)	Frequency/direction of appearance comparisons	Likert (varies by version)	Adolescents; Young adults	Proximal mechanism in social-media contexts	van den Berg, Thompson, Obremski-Brandon, and Coovert (2002) [19]; Karsay, Knoll, and Matthes [24]; Bonfanti, Melchiori, Teti, Albano, Raffard, Rodgers, and Lo Coco [25]
Self-discrepancy	Selves Questionnaire/discrepancy scoring	Actual–Ideal; Actual–Ought gaps (idiographic trait lists)	Idiographic; discrepancy indices	Adolescents; Adults	Risk/maintenance mapping to affect (shame/dejection; guilt/agitation)	Higgins [4]; Strauman, Vookles, Berenstein, Chaiken, and Higgins [26]; Mason, Smith, Engwall, Lass, Mead, Sorby, Bjorlie, Strauman, and Wonderlich [27]
ED psychopathology anchor	Eating Disorder Examination–Questionnaire (EDE-Q)	Global ED severity; subscales include restraint, eating concern, shape concern, weight concern; overvaluation indices	28 items; Likert	All ED diagnoses	Primary outcome: mediator with identity measures	Fairburn, Cooper, and Shafran [7]

Notes: Pair SCCS with AIDA in adolescents; consider SMI-ED when performing schema/mode work in adults. Report pre/post change (Δ) and minimal detectable change where available. Abbreviations: ED = Eating Disorder; AN = Anorexia Nervosa; SCCS = Self-Concept Clarity Scale; AIDA = Assessment of Identity Development in Adolescence; SMI-ED = Schema Mode Inventory—Eating Disorders; OBCS = Objectified Body Consciousness Scale; PACS/PACS-3 = Physical Appearance Comparison Scale (versions 1 and 3); EDE-Q = Eating Disorder Examination–Questionnaire.

**Table 2:** Clinical features of eating disorders across developmental stages.

Feature	Children (<12 Years)	Adolescents (13–18 Years)	Adults (>18 Years)
Typical onset	Increasing cases of early-onset AN and BED reported in preadolescence	Peak incidence of AN, BN, and BED	More chronic or relapsing presentations
Phenotype	Atypical AN (normal weight with severe complications); early BED with obesity	Classic AN with marked weight loss; BN with binge/purge; BED more frequent	Full DSM-5 syndromes, often comorbid with depression, anxiety, and personality disorders
Medical complications	Growth impairment, pubertal delay, bone mineral deficits, high medical instability	Amenorrhea, reduced bone density, electrolyte disturbances	Cardiovascular, metabolic, and gastrointestinal complications; osteoporosis
Psychosocial impact	Bullying, school avoidance, social withdrawal	Peer comparison, identity conflict, self-esteem vulnerability	Work, relationship, and role functioning impairment
Preferred treatment	Family-Based Treatment (FBT), pediatric medical monitoring	FBT, CBT-E adapted for adolescents, prevention programs	CBT-E, schema therapy, MBT, psychodynamic, or adjunctive methods

Abbreviations: AN = anorexia nervosa; BN = bulimia nervosa; BED = binge-eating disorder; FBT = family-based treatment; CBT-E = enhanced cognitive behavioral therapy; MBT = Mentalization-Based Therapy.

**Table 3:** Mapping mechanisms to interventions: identity-informed augmentation of standard ED care.

Mechanism/Maintenance Factor	Primary Intervention(s)	Key Techniques	Process Measures (Mechanism Capture)	Clinical Outcomes (Examples)
Overvaluation of weight/shape	CBT-E (focused)	Cognitive restructuring of overvaluation; pattern-breaking for checking/avoidance; regular eating	EDE-Q Overvaluation; body checking/body image avoidance scales	↓ EDE-Q global; symptom remission; BMI/weight restoration (when indicated)
Identity diffusion/low SCC	Narrative therapy, schema therapy, and values-based work (ACT)	Externalize ED identity; re-author self-story; schema/mode work; values clarification	SCCS; AIDA; SMI-ED	↑ SCC; ↓ drive for thinness/body dissatisfaction; ↑ quality-of-life
Self-objectification/appearance comparison	CBT-E + comparison-disruption; media-literacy; functionality appreciation	Reduce surveillance; guided comparison exposure; cultivate body functionality focus	OBCS (surveillance/shame); PACS-3	↓ body dissatisfaction; ↓ ED symptoms (EDE-Q)
Emotion/impulse dysregulation (binge/purge)	DBT-informed modules	Distress tolerance, emotion regulation, opposite action, and urge surfing	DERS; binge/purge urge logs	↓ objective binge/purge frequency; ↓ global ED psychopathology
Mentalization deficits (self/other)	MBT-ED (adjunct or program)	Mentalizing stance; affect-marked reflection; rupture repair; self-mentalizing	Reflective functioning measures; mentalization tasks	↓ weight/shape concern; improved alliance and functioning
Interoceptive/DMN disturbances	Interoceptive exposure; compassion-focused practices; neuromodulation (adjunct)	Signal labeling/exposure to hunger/fullness; compassion practices; rTMS/tDCS (selected cases)	MAIA (interoception); resting-state/task indices (research settings)	↑ satiety tolerance; ↓ ED cognitions; functional gains

Notes: Evidence levels vary (RCT vs. cohort vs. pilot). Select CBT-E focused vs. broad based on perfectionism, mood intolerance, interpersonal problems, and/or core low self-esteem. Abbreviations: CBT-E = Enhanced Cognitive-Behavioral Therapy for Eating Disorders; ACT = Acceptance and Commitment Therapy; MBT-ED = Mentalization-Based Therapy for Eating Disorders; DBT = Dialectical Behavior Therapy; DERS = Difficulties in Emotion Regulation Scale; SCC = Self-Concept Clarity; AIDA = Assessment of Identity Development in Adolescence; SMI-ED = Schema Mode Inventory—Eating Disorders; OBCS = Objectified Body Consciousness Scale; PACS-3 = Physical Appearance Comparison Scale—3; EDE-Q = Eating Disorder Examination–Questionnaire; MAIA = Multidimensional Assessment of Interoceptive Awareness; DMN = Default Mode Network; rTMS = Repetitive Transcranial Magnetic Stimulation; tDCS = Transcranial Direct Current Stimulation; BMI = Body Mass Index.

## Data Availability

No new data were created or analyzed in this study.

## Abbreviations

The following abbreviations are used in this manuscript:

ACT	Acceptance and Commitment Therapy
AFT	Adolescent-Focused Psychotherapy
AIDA	Assessment of Identity Development in Adolescence
AN	Anorexia Nervosa
BED	Binge-Eating Disorder
BMI	Body Mass Index
BN	Bulimia Nervosa
BPD	Borderline Personality Disorder
CBT-E	Enhanced Cognitive-Behavioral Therapy For Eating Disorders
CFT	Compassion-Focused Therapy
DBT	Dialectical Behavior Therapy
DBT-BED	DBT Adapted for Binge-Eating Disorder.
DERS	Difficulties in Emotion Regulation Scale
DMN	Default Mode Network
ED	Eating Disorder
EDE-Q	Eating Disorder Examination–Questionnaire
FBT	Family-Based Treatment
fMRI	Functional Magnetic Resonance Imaging
FPT	Focal Psychodynamic Therapy
GST	Group Schema Therapy
MAIA	Multidimensional Assessment of Interoceptive Awareness
MBT	Mentalization-Based Therapy
MBT-ED	Mentalization-Based Therapy for Eating Disorders
mPFC	Medial Prefrontal Cortex
OBCS	Objectified Body Consciousness Scale
PACS/PACS-3	Physical Appearance Comparison Scale (versions 1 and 3)
PCC	Posterior Cingulate Cortex
RCT	Randomized Controlled Trial
rTMS	Repetitive Transcranial Magnetic Stimulation
SASB	Structural Analysis of Social Behavior
SCC	Self-Concept Clarity
SCCS	Self-Concept Clarity Scale
SMI-ED	Schema Mode Inventory—Eating Disorders
SSCM-ED	Specialist Supportive Clinical Management—Eating Disorders
tDCS	Transcranial Direct Current Stimulation
Δ	Change (post-pre)
